# Impact of probiotic on anxiety and depression symptoms in pregnant and lactating women and microbiota of infants: A systematic review and meta-analysis

**DOI:** 10.7189/jogh.13.04038

**Published:** 2023-05-12

**Authors:** Kurvatteppa Halemani, Asha P Shetty, Latha Thimmappa, Alwin Issac, Sanjay Dhiraaj, K Radha, Prabhaker Mishra, Edlin Glane Mathias

**Affiliations:** 1 Sanjay Gandhi Post Graduate Institute of Medical Sciences, Lucknow, Uttar Pradesh, India; 2 All India Institute of Medical Sciences, Bhubaneswar, Odisha, India; 3 All India Institute of Medical Sciences, Kalyani, West- Bengal, India; 4 Manipal Academy of Higher Education, Manipal, Karnataka, India

## Abstract

**Background:**

Probiotics are non-invasive therapies composed of live bacteria and yeast. Administration of prebiotics improved the health status of pregnant and lactating women, as well as newborns. This review aimed to appraise the evidence concerning the effectiveness of probiotics on the mental health of pregnant women, lactating mother and the microbiota of the newborn.

**Methods:**

This systematic review and meta-analysis ascertained quantitative studies published in Medline (PubMed), Clinical Key, EMBASE, Cumulative Index to Nursing and Allied Health Literature (CINAHL), the Cochrane Library, and Google scholar. Two authors independently screened and extracted the data from the primary studies that analysed the efficacy of probiotics on the mental health of pregnant and lactating women and the microbiota of the newborn. We adopted Cochrane Collaboration guidelines and reported using the Preferred Reporting Items for Systematic review and Meta-Analysis (PRISMA) statement. The qualities of included trials were assessed by Cochrane collaboration's risk of bias tool (ROB-2).

**Results:**

Sixteen trials comprised 946 pregnant women, 524 were lactating mothers, and 1678 were infants. The sample size of primary studies ranged from 36 to 433. Probiotics were administered as interventions, using either a single strain of Bifidobacterium or Lactobacillus or a double-strain combination of Lactobacillus and Bifidobacterium. Probiotics supplementation reduced anxiety in pregnant (n = 676, standardised mean difference (SMD) = 0.01; 95% confidence interval (CI) = -0.28,0.30, *P* = 0.04, *I*^2^ = 70) and lactating women (n = 514, SMD = -0.17; 95% CI = -1.62,1.27, *P* = 0.98, *I*^2^ = 0). Similarly, probiotics decreased depression in pregnant (n = 298, SMD = 0.05; 95% CI = -0.24,0.35, *P* = 0.20, *I*^2^ = 40) and lactating women (n = 518, SMD = -0.10; 95% CI = -1.29,-1.05, *P* = 0.11, *I*^2^ = 60%). Similarly, probiotics supplementation improved the gut microbiota and reduced the duration of crying, abdominal distension, abdominal colic and diarrhoea.

**Conclusion:**

Non-invasive probiotic therapies are more useful to pregnant and lactating women and newborns.

**Registration:**

The review protocol was registered with PROSPERO (CRD42022372126).

The human digestive system or gastrointestinal tract consists of the mouth, oesophagus, stomach, small intestine, large intestine, and anus. The food is broken down in a series of steps with the help of digestive juices in the digestive system. As a result, the digestive system is a vital organ that breaks down food into small pieces and yields energy. The gut microbiota, or microbiome, is present in healthy people that improve gastrointestinal functions. While gut microbes assist in food absorption and digestion [1,2].

Probiotics consist of live bacteria and yeast that offer a range of health benefits. Probiotics enhance the gut microbiota and immunity and reduce inflammation and allergic reactions [3]. The gut microbiota is closely related to the central nervous system. Probiotics not only restore normal functions of the digestive system, but they also strengthen functions of other organs such as the nervous system, pituitary function and endocrine system [4].

The prevalence of anxiety, depression, and psychological upset is common in pregnant, perinatal, and postnatal women. However, mental health is the major contributing factor to low birth weight and preterm delivery. Therefore, balancing diet and a safe environment is important for pregnant and lactating women [5-7].

Antenatal nutrition and gestational age are important factors for establishing infant microbiota. In addition, several contributing factors altered the infant gut microbiota, such as preterm delivery, supplementary feeding use of drugs and dietary pattern [8-13]. Probiotic supplementation is a non-pharmacological, non-invasive treatment that benefits both nursing mothers and newborns. Results from an earlier study showed that probiotic supplements significantly reduced infant’s regurgitation, duration of cry and abdominal colic [14-16].

However, only a few systematic reviews and meta-analyses were conducted to evaluate probiotics’ effect on depression symptoms in adults [17,18], prenatal women [19] and perinatal and postnatal mothers. In addition, pooled the data from previous reviews unclear and retrieved from both randomized control trials (RCTs) and quasi-experimental studies. Therefore, this systematic review and meta-analysis aimed to evaluate the effectiveness of probiotics on mental health and microbiota of newborns.

## METHODS

The review has adopted Cochrane guidelines and reported using the Preferred Reporting Items for Systematic Reviews and Meta-analysis (PRISMA) [20]. The protocol has been registered with PROSPERO CRD42022372126.

A comprehensive search was conducted using a combination of MeSH terms or keywords connected to the PICO format. The search strategy was carried out using the following keywords: Antenatal women, parity, gravid, pregnant women, perinatal women, intra-natal, postnatal women, lactation mother, breastfeeding mother, neonates, newborn, infants, Lactobacillus acidophilus, probiotics, probiotic supplementation, microbiota, depression, mental health, anxiety, and afraid. Two authors (KH, AS) independently searched the online databases, namely MEDLINE (PubMed), Cumulative Index to Nursing and Allied Health Literature (CINAHL), EMBASE, Web of Science, SCOPUS, and Clinical Key, for RCTs published in the English language from January 2012 to October 2022. The included articles were screened, and duplicate articles were excluded based on review criteria. The reference tracking of relevant articles was manually searched for additional studies.

**Figure 1:**
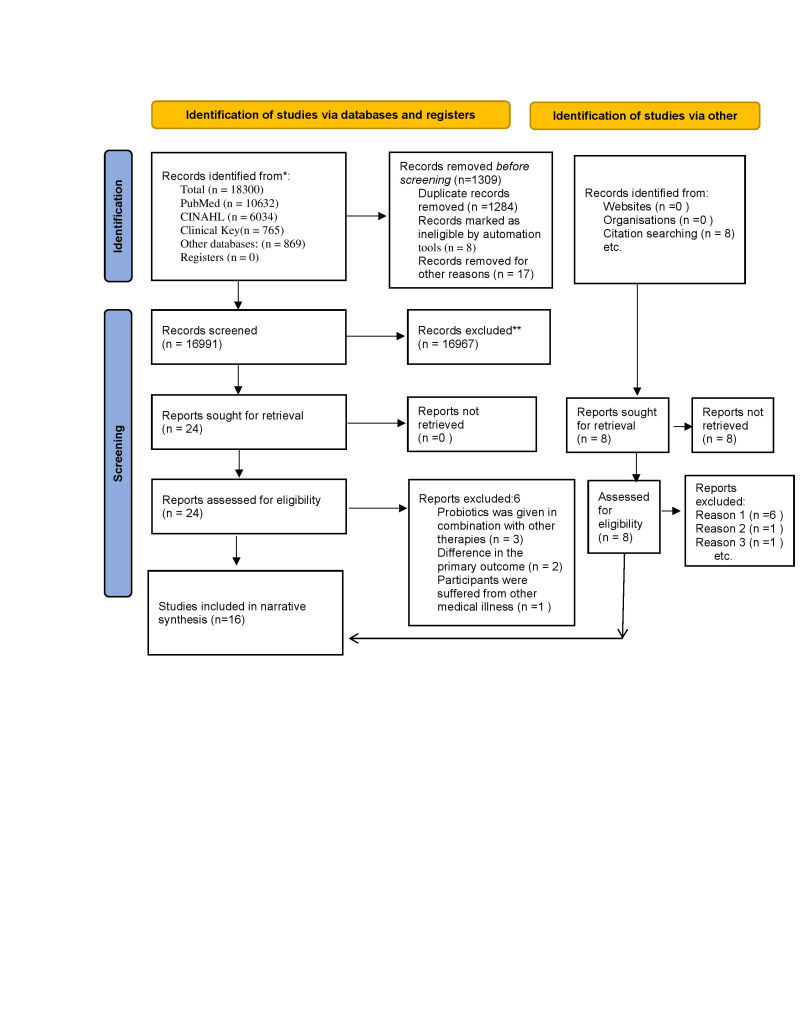
Flow diagram of study selection.

During the first stage of the search, a total of 18 300 items were identified, and eight studies from citation search with 1309 of them being removed before screening. The remaining studies’ methodology and abstract were screened. A total of 16 991 records, and 16 967 articles and eight citation search articles were excluded as they did not meet the review criteria. Further, we removed six articles due to the unavailability of the full text. Finally, 16 RCTs were included after fulfilling the review criteria: probiotic was the principle intervention for all primary studies published between 2012 and 2022 in the English language. Anxiety and depressions of pregnant and lactating women, as well as neonatal microbiota, were primary outcomes. The reasons for exclusion of primary studies were: probiotics combined with other supplementation and / or not evaluated depression and anxiety, difference in the primary outcome. The flow diagram of the study selection process is depicted in **Figure 1**.

**Figure 2:**
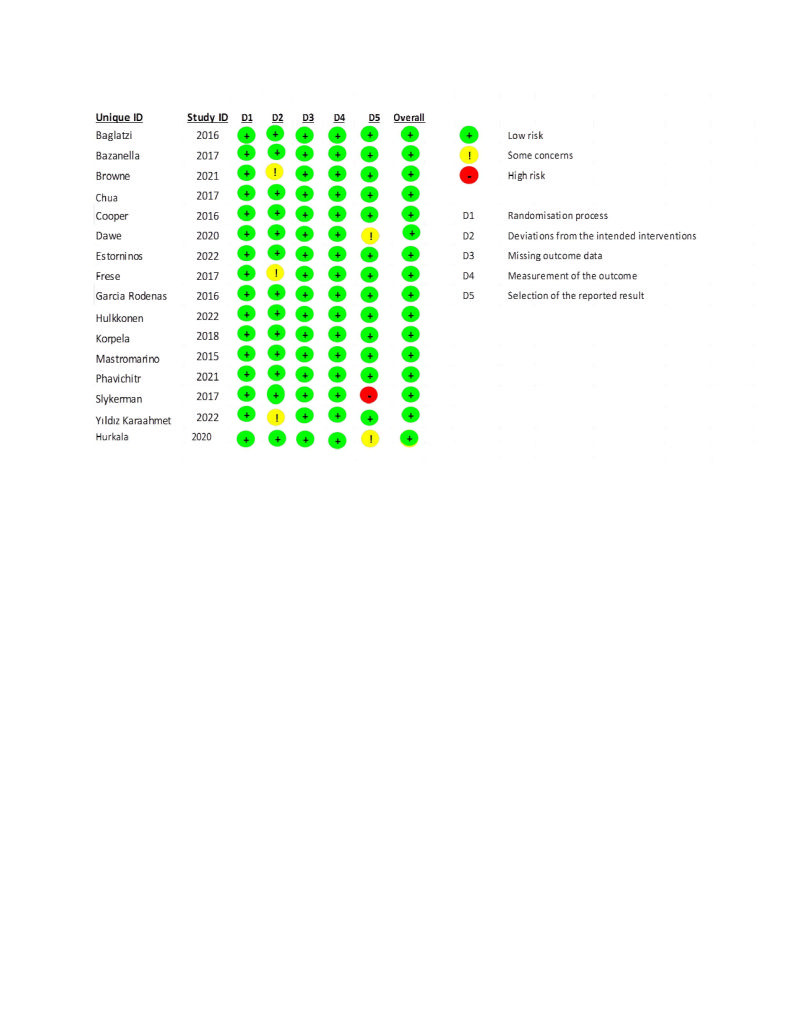
Risk of bias (ROB-2).

The Cochrane risk of bias tool-2 [21] was employed to assess the quality of the included RCTs. The details regarding the percentages across the included trials and judgments about each risk of bias item is depicted in **Figure 2**.

Two independent reviewers (KH) extracted the data from the primary studies, and any discrepancies in the initial judgments were confirmed by a third reviewer (LT). The data extraction form included the author's name, title, year of publication, country, study purpose, sample size, study design, analysis method, risk of bias, and major findings reported by the author(s).

### Statistics analysis

The meta-analysis was performed to examine the effects of probiotic supplementation among pregnant women, lactating mothers, and newborns. Anxiety, depression, and neonatal microbiota were the primary outcomes of the included studies evaluated between the probiotic and placebo groups. The effects of the probiotic intervention were calculated using a random-effects model to compute weighted and standardized mean differences with confidence intervals (CIs) of 95%. The heterogeneity of the included trials was analysed and classified as mild >50%, moderate 50%-75%, and high >75% using the *I*^2^ value.

## RESULTS

The pooled data of primary studies summarized under the following headings: authors name, year of publication, study design, sample size, type of intervention, and major findings (**Table 1**).

**Table 1:** Summary of included studies

Author Year, country	Research design	Sample size	Inclusion criteria	Exclusion criteria	Name of intervention and duration		Key findings
**Effect of probiotics on pregnant women and lactating mother**							
Slykerma, 2017, New Zealand	Double-blinded multi centre RCTs	Total = 433, probiotics = 212, placebo = 211	Pregnant English-speaking women with gestational ages from 12-16 weeks to six months postnatally. The study was carried out from December 2012 to November 2014 in Wellington and Auckland, New Zealand.	Pregnant or lactating women, have a serious medical illness, or are under the age of 16 y.	IG: L. rhamnosus HN001 dose of 6 × 10^9^ CFU per capsule. CG: Corn-derived maltodextrin. Duration: between 12-16 weeks gestation to six months postnatal.		There was a substantial difference between the two groups, with anxiety and depression being much lower in the probiotics group than in the placebo group. Probiotic therapy was found to be an effective treatment for anxiety and depression in pregnant and lactation women.
Hulkkonen, 2022, Finland	Double-blinded multi centre RCTs	Total = 343, E1: fishoil (FO) + placebo = 87, E2: probiotic + placebo = 96, E3: probiotics + FO = 93, C: control = 67	The first-trimester self-reported overweight pregnant woman registered between October 2013 – July 2017 at the University of Turku and Turku University Hospital in Turku, Finland.	Women having multiple foetal pregnancies with pregnancy spacing of less than 12 mo.	IG: L. rhamnosus HN001 and B. animalis ssp. lactis 420 CG: microcrystalline cellulose. Duration: from first trimester.		Probiotics and FO have mild to moderate antidepressant properties. Inadequate nutrition intake was most common causes of depression and anxiety. Different modes of delivery have significant statistical differences in EPDS scores (*P* = 0.018).
Browne, 2021, Netherland	RCT blinded research design	Total = 40 IG (probiotic) = 20 CG (placebo) = 20	Pregnant women over the age of 18, have a history of moderate depression or anxiety in their second or third trimester. All cases were reported at Radboud University Medical Center in Nijmegen, the Netherlands.	Pregnant women with multiple foetuses, drug addiction, chronic medical illness, suicidal behaviour, and bipolar psychiatric illnesses.	IG: oral probiotic multispecies (ecologic barrier; 2.5 × 109 CFU / g; daily dosage 2 g;), bifidum W23, B. lactis W51, B. lactis W52, lactobacillus acidophi- lus W37, L. brevis W63, L. casei W56, salivarius W24, L. lactis W19 and L. lactis W58. CG: indistinguishable regarding colour, taste, and smell (Winclove). Duration: 26-30 weeks.		Probiotics are a powerful intervention for improving cognitive development. As a result, it has no effect on anxiety, depression, or pregnancy-related symptoms.
Dawe, 2020, New Zealand	RCT two factorial design	Total = 130, probiotics = 115, placebo = 115	A singleton 12-17 weeks pregnant with a BMI greater than 30 kg / m^2^ recruited between April 2015 and June 2017 at department of Obstetrics and Gynecology, Faculty of Medical and Health Sciences University of Auckland.	Gestational diabetic women with HbA1c more than normal. Congenital abnormalities in foetuses.	IG: L. GG and B. lactis BB12 (Chr. Hansen A/S, Hoersholm, Denmark), at a minimum dose of 6.5 × 10^9^ CFU CG microcrystalline cellulose and dextrose anhydrate (Chr. Hansen A / S, Hoersholm, Denmark). Duration: 12-17 weeks.		Probiotics was not significant to improve the mental health of pregnant women.
**Effect of probiotics on lactating mother and newborn**							
Mastromarino, 2015, Italy	Double-blinded multi centre RCTs	Total = 66, probiotics = 33, placebo = 33	Pregnant women or breastfeeding women age ranged from 18-45 y registered at the University of Bari in Italy, from April 2011 to December 2013.	High risk pregnancy and chronic illness.	IG: mother probiotics mixture such as 9 × 10^11^ of VSL# L. acidophilus, L. plantarum, L. paracase, L. debrueckii and bulgaricus, B. infantis and S. thermophilus. CG: corn starch. Durartion: 36 weeks to one month postnatal.		The probiotics group, especially the normal vaginal birth increased the amount of lactobacilli and biofobacteria in their colostrum and mature breast milk.
Korpela, 2018, Finland	Double-blinded multi centre RCTs	Total = 422, probiotics = 199, placebo = 223	The gestational age ranges from 37 to 42 weeks. Pregnant women with allergy had vaginal or C-section births. Mothers with allergy had exclusively breastfed.	Preterm babies. The infants with congenital malformation.	IG: mother – twice day, 5 *×* 10^9^ L.GG, 5 *×* 10^9^ CFU L. rhanmosus LC705, 2 *×* 10^8^ CFU. P freudenreidii ssp, shermanii JS. Newborn: probiotic mixture given same as mother but dose 0.8 g of GOS. CG: microcrystalline cellulose. Duration: from eighth month of gestation till sixth month postnatal.		The mixture of probiotic like B. breve and L. rhamnosus given with breast feeding significantly support the neonate microbiota.
Yıldız Karaahme, 2022, Istanbul	Single blinded RCTs	Total = 36, probiotics = 18, placebo = 18	Infant aged 30-60 d with complaints of persistent crying. The newborn was delivered vaginally at 37-42 weeks and was fed 8-10 times per day.	Gastro intestine illnesses affect either the mother or the child. Mother who already takes probiotics.	Probiotics 5 × 106 actiregularis contents mixture administered to mother with babies for 15 d. Duration: from second trimester till postnatal days.		Duration of cry and intensity lessen (*P* = 0.00). Increased the variety of bacterial in the faces (*P* = 0.017).
**Effect of probiotics on newborns**							
Baglatzi, 2016, Switzerland	Double-blinded multi centre RCTs	Total = 198, probiotics = 77, placebo = 77, Reference group 44	Baby weighs 2.5 kg to 4.5 kg, delivered by C-section, and mother was discontinued breastfeeding 24 h after delivery. Breastfeeding for given at least four months in control group.	Rotarix vaccination was administered to the newborns. Mother is already involved in other clinical trials.	IG: 10^7^ CFU / g mix with B. bifidum BF3, B. breve BR3, B. longum BG7, B. longum given along infant formula. CG: breastfeeding. Duration: intervention give between birth to six months.	The infant in the intervention group detected B. lactis at a higher percentage (85%) than that of the control group (47%). However, newborns delivered by C-section showed lower levels of B. lactis. Although, B. lactis supplementation may improve gut microbiota.	
Cooper, 2016, South Africa	Double-blinded multi centre RCTs	Total = 421, probiotics = 207, placebo = 214	Healthy infant with HIV-infected mother who chose to breastfeed. The gestational age varied from 37 to 42 weeks. Newborn weight range from 2.5 to 4.5 kg and less than three days of life.	Congenital malformation or babies received antibiotics during first 72 h of life. Parents had previously taken part in research studies.	IG: 1 × 10^7^ CFU / g of B. animalis subsp lactis CNCM I-3446 and 5.8g / 100g of a combination of bovine milk-derived oligosaccharides (BMOS). CG: formula feed. Duration: intervention between birth and six months.	The bifidogenic impact of mixed formula with probiotics was boosted in newborns. The use of newborn formula has the same effect in normal and C-section births. Thus, bifidobacteria operate as probiotics that are safe in newborn or immunocompromised newborn.	
Garcia Rodenas, 2016, Switzerland	Double-blinded multi centre RCTs	Total = 40, probiotics = 20, placebo = 20	Full-term infants with more than 72 h on exclusively breastfed. Parents were agreed to participate in the study.	Congenital malformation or chromosomal disease. Mother has taken Antibiotics or prescribed to their child.	IG: The combine infant formula with 10^7^CFU / L L. reuteri DSM BF3, B. breve BR3following 72 h of birth. CG: formula feed. Duration: between 72 h after delivery and six months.	Probiotic supplements in formula feed improved L. reuteri levels in infants. L. reuteri, on the other hand, promotes the growth of L. spp and microbiota, including the early development of microbiota in newborns during vaginal delivery.	
Bazanella,2017, Germany	Double-blinded multi centre RCTs	Total = 106, probiotics = 48, placebo = 48,other = 9	Healthy-term newborns delivered vaginally or by C-section. Continue on breastfeed or formula feeding.	Premature newborns, high-risk pregnancies, and serious maternal sickness. Antibiotic treatment and body mass index BMI<18.5 or >30.	IG: Whey based formula of 10^7^ CFU / g of B. bifidum, B. breve, B. longum. *CG:* formula feed. Duration: intervention between birth and six months.	Bifidobacteria supplements reduced Bacteroides and Blautia spp among newborns in intervention group. However, the gut microbiota did not change.	
Chua, 2017 Thailand	Double-blinded multi centre RCTs	Total = 183, E1-probiotics = 52, E2-synbiotics = 51, E3-placebo = 80	Healthy babies were recruited in Singapore between June 2011 and April 2013.	Congenital deformities or serious disease were not taken into account.	E1: The mixed formula feed with 0.8 g / 100 mL scGOS/lCFOS E2: *B.* breve M-16V (synbiotic) or scGOS / lCFOS (prebiotic). CG: formula feed. Duration: after birth 24-72 h till four months.	Prebiotics (E1) and synbiotics (E2) boosted gut emulation in newborns delivered by C-section, but not in vaginal delivery.	
Frese, 2017, USA	RCTs	Total = 66, probiotics = 34, synbiotics = 32	The exclusive breastfeeding baby at least three months age, not born through C-section.	Infants on antibiotics, acute respiratory problem. Mother had breast surgery or metabolic issues within five years.	IG: breastfeeding plus capsule composed of 1.8 *×* 10^10^ CFU of B. longum sub. *CG:* formula feed. Duration: first weeks to one month after birth.	Enterobacteriaceae, clostridiaceae, erysipelotrichaceae, pasteurellaceae, micrococcaceae, and lachnospiraceae favorable in intervention group and decreased endotoxin levels.	
Estorninos, 2022, Philippines	Double-blinded multi centre RCTs	Total = 226, probiotics = 114, placebo = 112, other = 70	Healthy singleton with normal gestational – with postnatal age 21-26 d. On exclusive BF. Weight for height and head circumference Z scores: -3 and +3.	Congenital malformation, prenatal or postnatal complications. Admission of infants to neonatal critical care units.	IG: The mixture of infant formula composed of 7.2 g / L bovin milk derived oligosaccharides. CG: formula feed. Duration:26 d to six months after birth.	Infants fed milk-derived oligosaccharides MOS formula had a higher intestinal immune response than the control and reference groups. Infants undergoing C-section have better gut microbiota than those undergoing vaginal birth.	
Phavichitr,2021, Thailand	Double-blinded multi centre RCTs	Total = 290, probiotics = 81, synobiotics = 82, reference group = 84, other = 43.	Healthy normal gestational age. On exclusive formula feeding with postnatal age 43-65 d.	Malnourished babies under weaning or taking antibiotics. Those who suffer from serious gastro intestinal illnesses.	IG1: 0.8 g / 100 ml, ScGOS/lcFOS and B. breveM-16v (1-104 CFU / 100 ml IG2 = 0.8 g / 100 ml, scGOS / lcFOS and B. breve M-16v (1-106 CFU / 100 ml). CG: formula feed. Duration: between birth and six month after birth.	Synbiotic formula increased bifidobacteria proportions while decreasing C. difficile. The intervention groups had lower stool PH and higher amounts of L-lactate and acetate than the control group.	
Hurkala, 2020, Poland	RCT	Total = 148, probiotics = 71, placebo = 77	Normal healthy infants delivered by caesarean section Apgar scale: 8-10 points, suitable gestational mass >2.5 kg. Parents given informed written consent.	Parents were not given informed consent.	IG: oral capsule containing 2106 CFU / day. Bifidobacterium breve PB04 and lactobacillus rhamnosus KL53A. CG: formula feed / breastfeed. Duration: between birth and six month after birth.	Supplementation of L. rhamnosus and B. breve strains immediately after birth that help to increases numbers of lactobacilli and bifidobacteria in infants.	

RCT – randomized control trial, IG – intervention group, CG – control group, L – lactobacillus, B – bifidobacterium, CFU – colony forming units, BF – breast feeding, y – years, mo – months, d – days, h – hours

### Systematic review

This systematic review and meta-analysis comprise 946 pregnant women [22-25], 524 lactating mothers [26-28], and 1678 newborns [29-38]. Sample size of primary studies ranged from 36 [34] to 433 [24]. All original trials adopted double-blinding [22,23,25-27,29-36] except two studies [37,38], reported worldwide, namely New Zealand [22,24,29,30], Finland [25,27], Thailand [31,32], Philippian [33], the Netherlands [23], Italy [26], Istanbul [34]], South Africa [35], Germany [36], USA [37]] and Poland [38].

### Probiotics administered in pregnant and lactating women

Four trials (n = 946) assessed the effectiveness of probiotics on anxiety and depression among pregnant women [22,23,25] and lactating mother [24]. Probiotics were administered in the first trimester [25], second trimester [22,24], and third trimester [23]. Probiotics were administered to intervention groups composed of L. rhamnosus HN001 [24], L. rhamnosus HN001 and B. animalis ssp. lactis 420 [25], bifidum W23, B. lactis W51, B. lactis W52, Lactobacillus acidophi- lus W37, L. brevis W63, L. casei W56, salivarius W24, L. lactis W19 and L. lactis W58 [23] and L. GG and B. lactis BB12 [22]. Supplementation of probiotics decreased the anxiety and depression in pregnant and lactating women [24]. Also, it reduced mild depression [25] and improved cognitive functions in pregnant women [23] (**Table 1**).

### Probiotics administered in lactating mother and newborn

Three studies (n = 524) assessed the effectiveness of probiotics among lactating women and their newborns [26,27,34]. The following probiotics combinations used as interventions such as 9 X 10^11^ of VSL, L. acidophilus, L. plantarum, L. paracase, L. debrueckii and bulgaricus B. infantis & S. thermophilus [26] 5 X 10^9^ L.GG, 5 X 10^9^ CFU L. rhanmosus LC705, 2 x 10^8^ CFU P freudenreidii ssp, shermani JS [27] and 5 × 10^6^ actiregulari [34]. Compared to the control group intervention group increases lactobacilli and bifidobacteria in colostrum and mature breast milk [26] improves gut microbiota [27] and reduces the duration of cry [34] (**Table 1**). Probiotics lower the risk of anxiety and depression in pregnant and lactating women.

### Probiotics supplementation in newborns

The remaining nine trials contain full-term, healthy newborns with normal birth weight (n = 1678) and assess the probiotic therapy on neonatal microbiota [23,29-33,35-38]. Three of the nine trials reported newborns delivered via caesarean section (C-section) [29,32,38], while the other six trials reported both C-section and vaginal deliveries [30,31,33,35-37] (**Table 1**). Probiotics boost the gut microbiota of newborns.

The intervention group received probiotic supplementation in addition to regular breastfeeding for six months. The probiotics are composed of B. bifidum BF3, B. breve BR3, B. longum BG7, B. longu [29], L. rhamnosus and B. breve [30,32,36,38], Bovin milk derived [35], oligosaccharides [33], B. longum [37] sub. calculated based on an infant’s weights. The intervention group had higher percentages B. lactis [29], the early development of gut microbiota [30,33,35] or gut emulation [31], decreased endotoxin levels [37] or lower stool Ph and high amounts of L-lactate and acetate [32,38].

### Probiotics strain, dosages

For pregnant women, the single probiotic strain used was Lactobacillus [24], and the combined strain was Lactobacillus and Bifidobacterium [22,23,25]. Probiotics provided were based on species namely Bifidobacterium animalis [22,25]. Probiotics provided were based on species namely Bifidobacterium animalis, [23] or based on genus lactobacillus stated different species Lactobacillus L. rhamnosus [22,24,25] and L. brevis [23]. Probiotic, dosages were expressed in colony forming unit (CFU) per millilitre [30-32], CFU per kilogram [29,35,36] and CFU per day [22,24-27,37,38] (**Table 1**).

For newborn probiotics, a single strain of the Bifidobacterium [29,37], Lactobacillus [30], or a combination of Lactobacillus and Bifidobacterium [26,27,36,38]. Similarly, probiotics interventions were based on species, namely Bifidobacterium breve [26,27,32,36,38] Bifidobacterium longum [26,36,37] B. animalis. Based on genus lactobacillus using different species Lactobacillus namely L. acidophilus, L. delbrueckii subsp, L. plantarum, L. reuteri [26,27,36,38] (**Table 1**).

### Meta-analysis

Only four primary studies reported numerical data on mental health among pregnant and lactating women. Therefore, we selected these trials for the meta-analysis.

### Effect of probiotics on anxiety among pregnant and lactating women

**Figure 3:**
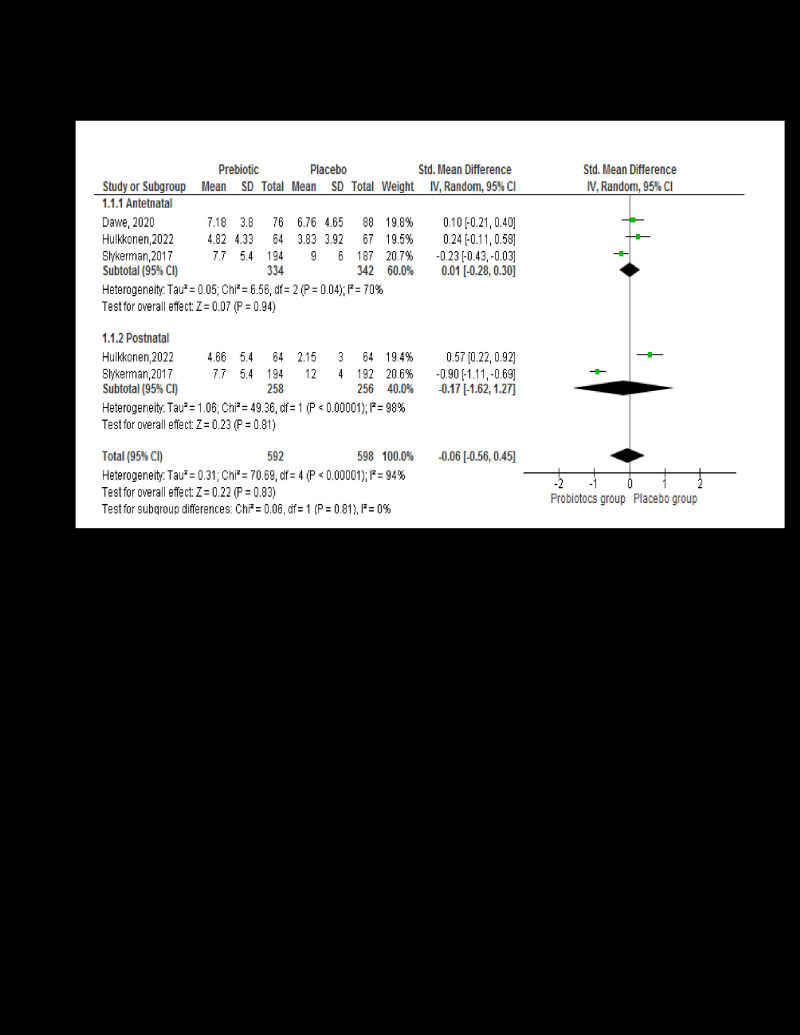
Effect of probiotics on anxiety.

Three studies reported the effect of probiotics supplementation on anxiety among pregnant women (n = 676, SMD = 0.01; 95% confidence interval (CI = -0.28,0.30, *P* = 0.04, *I*^2^ = 70) [22,24,25]. Similarly, two studies reported on probiotic usage among lactating women (n = 514, SMD = -0.17; 95% CI = -1.62,1.27, *P* = 0.00, *I*^2^ = 98%) [24,25] (**Figure 3**).

### Effect of probiotics on depression among pregnant and lactating women

**Figure 4:**
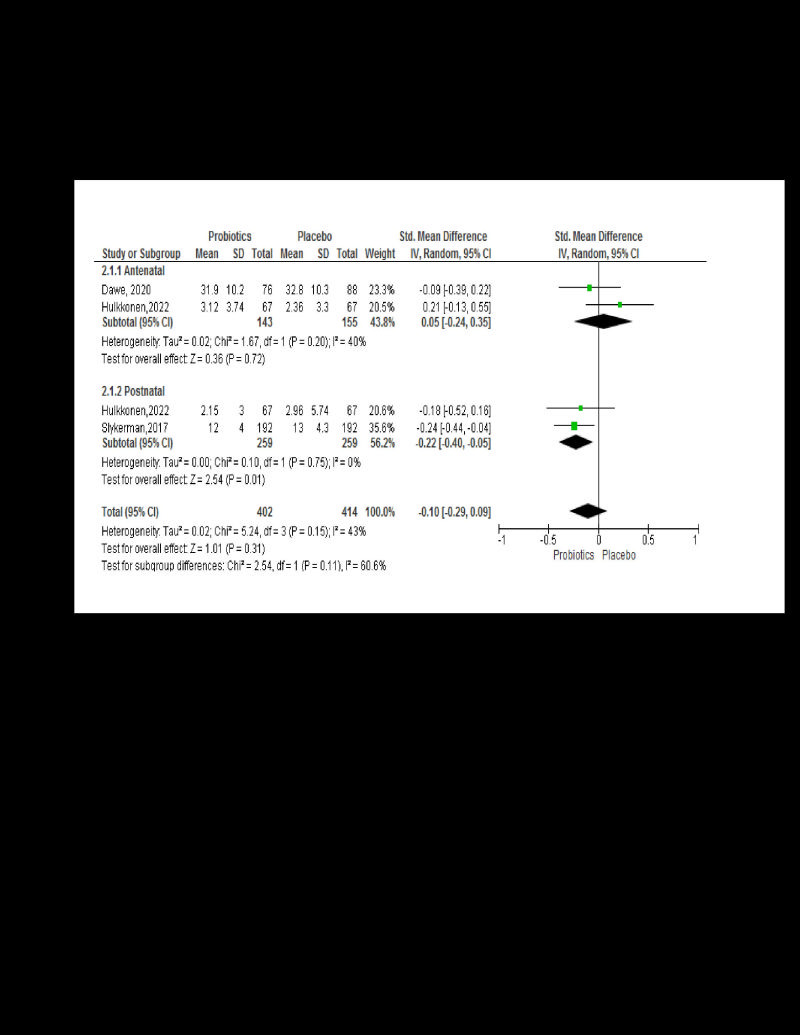
Effect of probiotics on depression.

Two studies the impact of probiotic supplements on pregnant women’s depression (n = 298, SMD = 0.05; 95% CI = -0.24,0.35, *P* = 0.20, *I*^2^ = 40). Similarly, two studies documented among breastfeeding mothers (n = 518, SMD = -0.10; 95% CI = -1.29,-1.05, *P* = 0.11, *I*^2^ = 60%) [22,25]. However, subgroup analysis was not found to be significant between experimental and control groups (**Figure 4**).

## DISCUSSION

This systematic review and meta-analysis evaluated the efficiency of probiotic supplementations for pregnant and lactating women and infant's microbiota. Supplementation of probiotics in pregnant women has improved mental health [19,39]. There is strong evidence that probiotics benefit the gut and nervous systems, known as the “gut-brain axis” [40,41]. The role of probiotics in alleviating mood and cognition problems is still controversial. Several pieces of evidence support the claim that probiotics benefit both gut and brain health. In addition, recent studies have proved that there is a strong link between the digestive system and the nervous system [42].

Lactobacillus and Bifidobacterium are probiotic bacteria that have been shown to improve immune system function, respond to the formation of short-chain fatty acids, reduce inflammation, and boost gastric emptying through the action of mucosal receptors [43].

Probiotics improved the gut microbiota of neonates and reduced abdominal distention, colic and cry duration [44]. Nevertheless, some studies stated probiotics species, namely Lactobacillus, Bifidobacterium, Lactobacillus acidofilus, reuteri, and Streptococcu [45,46]. The dosage of probiotics supplementation was given as follows: 10^10^ cells for 21 days, 10^10^ cells for 14 days, and 10^9^ cells for 21 days [47]. Similarly, two studies reported dosages of probiotics 5 × 10^7^, 1.5 × 10^8^, 4.5 × 10^8^, 1.4 × 10^9^, 4.2 × 10^9^ given at first, second, third, fourth and five weeks, respectively [48,49]. Probiotics help in the digestion of newborns and reduce abdominal distension, colic, constipation, necrotizing enterocolitis, and diarrhoea. Specific probiotic strains treat paediatric diseases and are available in capsules, nutrition, and infant formula [50-52].

The main strength of our review is that we included double-blinded, randomized control trials. We also summarized the dosage, strain, and species of probiotics. Our systematic review and meta-analysis revealed that probiotic supplementation is a non-invasive therapy that can be used routinely in prenatal and postnatal women and newborns to improve the physical and mental well-being and microbiota of newborns [53]. Probiotics, prebiotics and synbiotics supplementation reduced prenatal depression [54], newborn abdominal colic [45,55] and regurgitation [55].

Various biomarkers and genetic methods are used to create distinct probiotic strains and species to increase neonatal gut microbiota colonisation. As a result, more randomized control trials are needed to investigate the specific probiotic strains used to understand the effects on pregnancy, lactating mothers, and newborns.

There were significant limitations; some studies included C-sections and vaginal births. Original studies did not report numerical data on primary outcomes, especially neonates’ microbiota. Lastly, anxiety and depression decreased in the probiotics group, but the primary studies were unclear about other extraneous variables that may affect mental health in pregnant and lactating women.

## CONCLUSION

Non-invasive therapies, such as probiotics, are more useful to pregnant and breastfeeding women and newborns. Administration of probiotics enhanced the gut microbiota, particularly newborns. The microbiome promotes gut colonization in newborns, influencing digestion and nutrition absorption. Henceforth this study provides strong evidence of the importance of probiotics for prenatal, postnatal, and newborn health.
